# Encapsulated Papillary Carcinoma of the Male Breast With a Mixed Invasive Component: A Report of a Rare Case

**DOI:** 10.7759/cureus.98665

**Published:** 2025-12-07

**Authors:** Nektarios Ntalakos, Maria Arnaouti, Evdokia Arkoumani

**Affiliations:** 1 Department of Pathology, Saint Savvas Anticancer Hospital of Athens, Athens, GRC

**Keywords:** breast cancer pathology, breast papillary lesions, encapsulated papillary carcinoma with invasion, male breast carcinoma, primary breast malignancy

## Abstract

Male breast carcinoma (MBC) is an uncommon disease, representing only a very small fraction of breast cancer diagnoses. Encapsulated papillary carcinoma (EPC), a distinct subtype, is even more uncommon in men. EPC may coexist with invasive components, requiring careful evaluation to differentiate it from benign papillary proliferations and metastatic malignancies. A 69-year-old man presented with a painless breast mass. Mammography revealed a well-circumscribed, partially cystic lesion. Left mastectomy with axillary sampling was performed. Histology showed EPC with a mixed invasive component composed of invasive carcinoma of no special type (NST) and invasive papillary carcinoma, measuring 23 mm. The tumor was estrogen receptor (ER)-positive, progesterone receptor (PR)-positive, human epidermal growth factor receptor 2 (HER2)-low, with a Ki-67 proliferation index of 25%. All six lymph nodes were negative for metastasis (pT2N0). The differential diagnosis of EPC in men includes gynecomastia, benign papillary lesions, solid papillary carcinoma, invasive papillary carcinoma, and metastases from prostate, thyroid, or gastrointestinal carcinomas. Accurate diagnosis requires integration of histology, myoepithelial markers, and site-specific immunohistochemistry. Treatment principles for MBC mirror those of female breast cancer and include mastectomy, endocrine therapy (tamoxifen), radiotherapy, and systemic therapy as indicated. Genetic counseling is recommended due to the high prevalence of germline mutations. This case highlights the rarity of EPC with a mixed invasive component in men and underscores the importance of comprehensive morphologic and immunophenotypic evaluation. Increased reporting of such cases will improve understanding and management of male breast malignancies.

## Introduction

Male breast carcinoma (MBC) is an uncommon malignancy, accounting for <1% of all breast cancers and <0.1% of cancer-related deaths in men [1,2]. Its incidence worldwide remains below one case per 100,000 men annually, with notable geographic differences, ranging from approximately 1.24 cases per 100,000 man-years in Israel to 0.16 per 100,000 in Thailand [1,2]. Epidemiologic studies indicate a higher incidence among Black men, who tend to present at a younger age and with more advanced disease, contributing to increased disease-specific mortality compared with other racial groups [1,2]. Conversely, lower rates have been documented in Japanese and Southeast Asian populations [1,2].

Biologically, MBC exhibits distinct molecular patterns across ethnic groups. In non-Hispanic White men, approximately 83% of tumors are hormone receptor-positive, 15% are human epidermal growth factor receptor 2 (HER2) positive, and fewer than 3% are triple negative [2]. Non-Hispanic Black and Hispanic men show higher frequencies of HER2-positive and triple-negative phenotypes, although overall survival does not appear to differ significantly among racial groups [1,2].

The rising incidence of MBC likely reflects population aging and improved detection, although additional environmental or hereditary factors contribute [2]. Unlike female breast cancer, which demonstrates a bimodal age distribution, MBC typically presents later in life, with a median age of 68 years [1,2]; however, cases in younger men and adolescents have been reported [1,2]. U.S. population data also show a decline in both incidence and mortality between 1996 and 2005, with mortality decreasing by 28% in men during this period [2].

Encapsulated papillary carcinoma (EPC) is a rare variant of papillary breast carcinoma characterized by delicate fibrovascular cores lined by neoplastic epithelial cells confined within a cystic space and surrounded by a fibrous capsule [1]. A hallmark of EPC is the absence of myoepithelial cells within the papillae and along the capsule - an important feature distinguishing it from benign papillary lesions [1,2].

EPC predominantly affects postmenopausal women, with peak incidence in the seventh decade of life [1-3]. Although rare, EPC represents a disproportionately higher fraction (3.5%-13.7%) of breast cancers in men compared with the prevalence of invasive ductal carcinoma (IDC) or ductal carcinoma in situ (DCIS) in the male population [2,3]. Approximately half of EPCs arise centrally and may present with nipple discharge, which is bloody in up to one-third of cases [1-3]. Tumor size is variable, ranging from a few millimeters to >10 cm, and although many patients present with a palpable mass, some remain asymptomatic, highlighting the need for proper imaging and histological evaluation [1-3].

## Case presentation

A 69-year-old male with a medical history of hypertension and hyperlipidemia presented with a painless mass in the left breast, persisting for approximately three months. On physical examination, the mass measured about 7 cm in greatest dimension, without associated nipple retraction, ulceration, or discharge.

**Figure 1:**
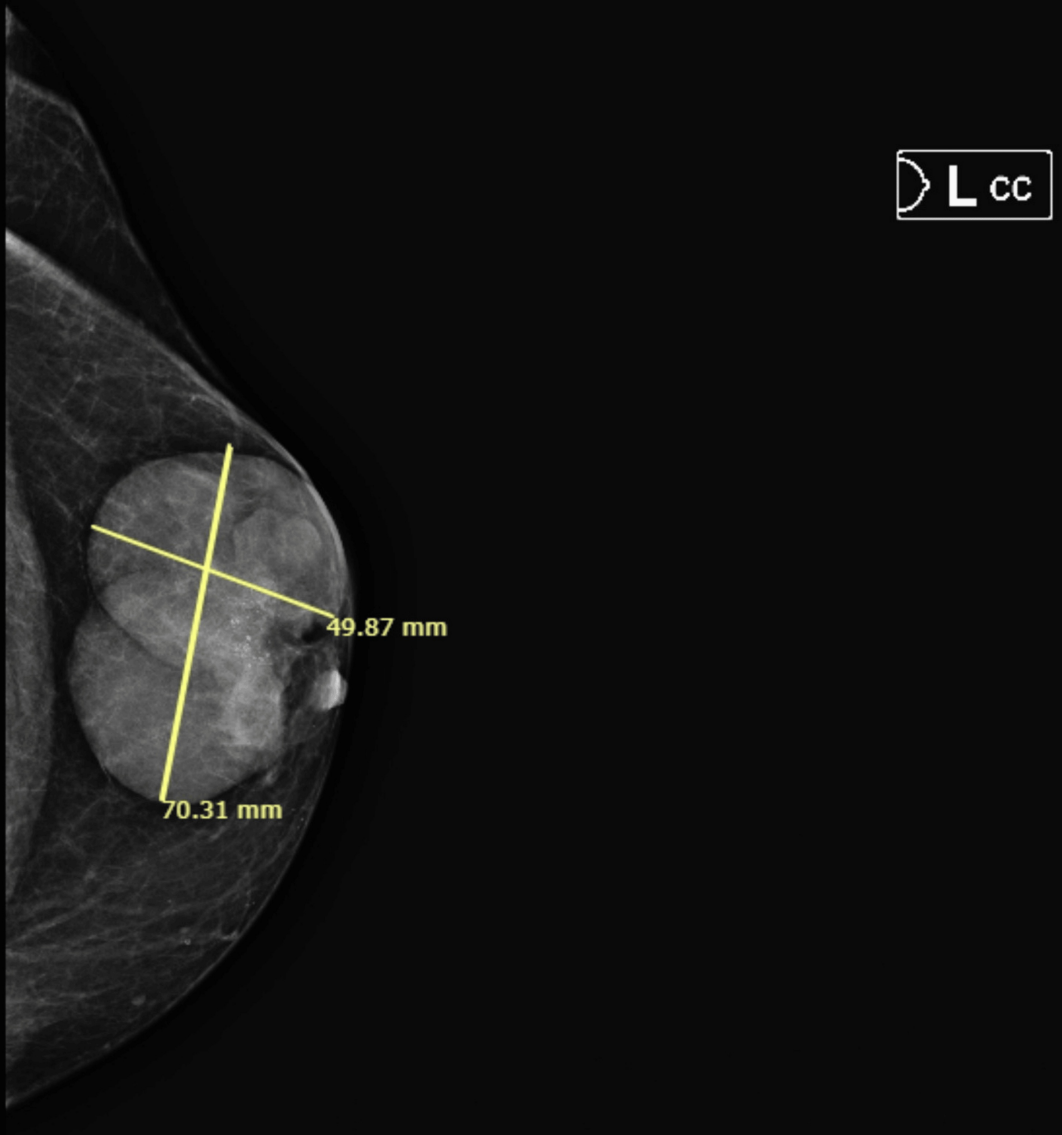
Mammographic appearance of the left breast mass (craniocaudal projection). Craniocaudal mammographic view of the left breast demonstrating a well-circumscribed, oval, retroareolar mass measuring approximately 7.0 × 5.0 cm. The lesion shows smooth margins and homogeneous density, without associated microcalcifications or architectural distortion, with findings suggestive of a partially cystic, encapsulated neoplasm.

Mammography of the left breast (craniocaudal projection) revealed a well-circumscribed, oval, retroareolar mass measuring approximately 7.0 × 5.0 cm. The lesion demonstrated smooth margins and homogeneous density, with no microcalcifications or architectural distortion. These findings were suggestive of a partially cystic, encapsulated lesion (Figure 1).

Based on the imaging and clinical findings, a left mastectomy with axillary lymph node sampling was performed, and the specimens were sent to the Pathology Department for further studying.

Gross examination of the mastectomy specimen showed a cystic mass containing blood-serous fluid with a central solid component, located in the retroareolar region and measuring up to 6 cm in greatest dimension.

**Figure 2:**
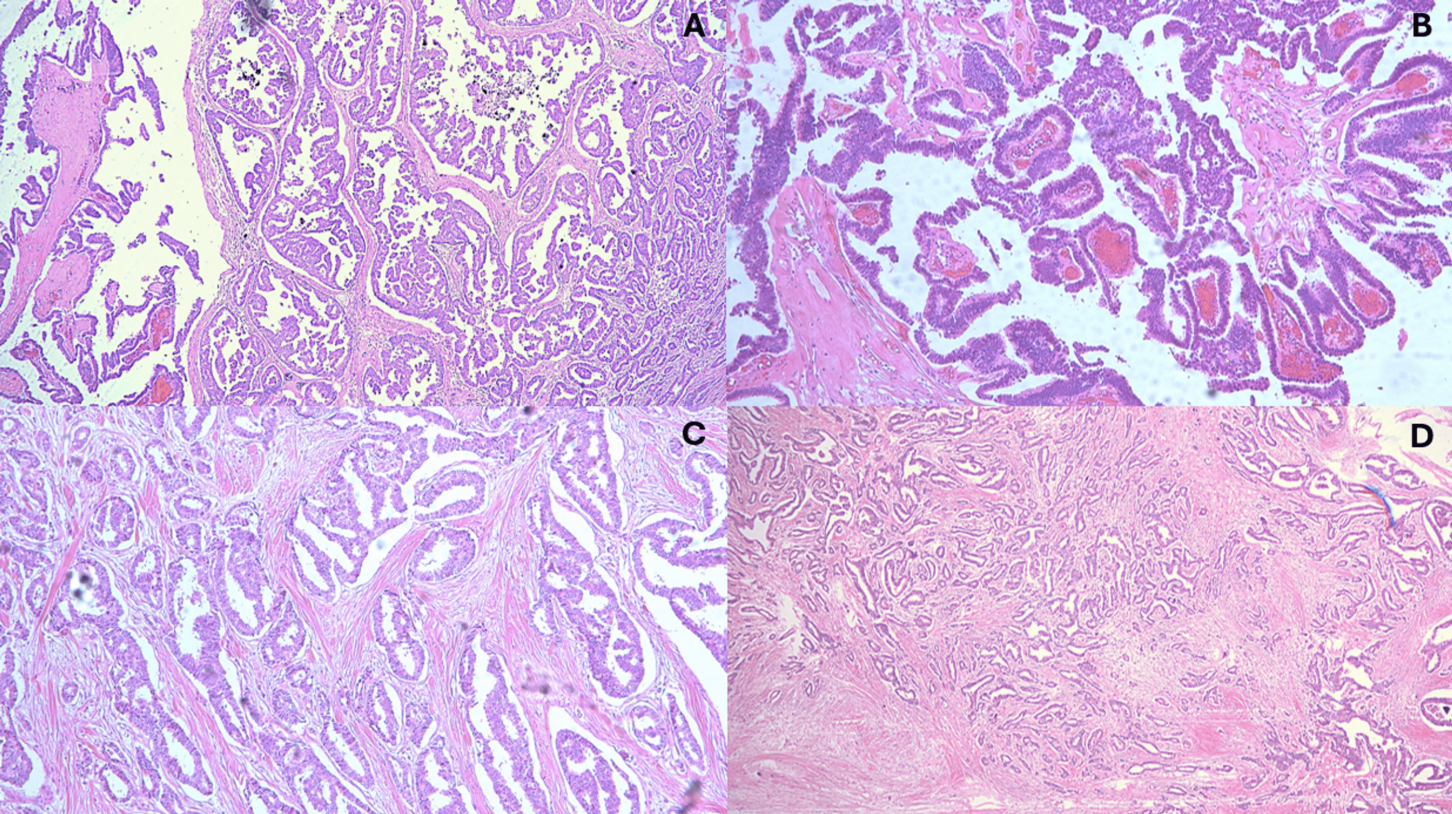
Histopathological features of the encapsulated papillary carcinoma (EPC) with a mixed invasive component. (A) Low-power view demonstrating the overall architecture of the lesion, showing an encapsulated papillary carcinoma with adjacent invasive components. (B) Higher-power view of the EPC component, characterized by delicate fibrovascular cores lined by neoplastic epithelial cells within a well-defined cystic space. (C) Invasive papillary carcinoma component, exhibiting infiltrative papillary structures with fibrovascular cores invading the surrounding stroma. (D) Invasive carcinoma of no special type (NST), showing irregular infiltrative nests and tubular structures within a desmoplastic stromal reaction.

Microscopically, the lesion exhibited features consistent with encapsulated papillary carcinoma of the male breast, with an associated mixed invasive component composed of no special type (NST) and IPC (Figure 2).

The invasive component measured 23 mm in greatest dimension and consisted of approximately 20% invasive NST (Grade 2) and 80% IPC (Grade 2). No evidence of lymphovascular invasion or dermal infiltration was identified.

Immunohistochemical analysis demonstrated strong estrogen receptor (ER) and progesterone receptor (PR) positivity, HER2-low expression, and a Ki-67 proliferation index of 25%.

Examination of the axillary lymph node specimen revealed six lymph nodes, all negative for metastatic involvement. Based on these findings, the tumor was staged as pT2N0 according to the American Joint Committee on Cancer (AJCC) 8th edition staging system.

## Discussion

Etiopathogenesis

MBC develops through a multifactorial interplay of genetic, hormonal, and environmental factors. Age is the most significant non-modifiable risk factor, with incidence rising after the sixth decade of life, although younger men may also be affected [4]. A positive family history is present in up to one-third of cases, and pathogenic germline variants, especially in BRCA2, but also BRCA1, CHEK2, PALB2, PTEN, and TP53-substantially increase risk [4-7]. BRCA2 mutation carriers have a markedly elevated relative risk (up to 44-fold), and approximately 10% may develop breast cancer during their lifetime [4].

Hormonal imbalance plays a central role in male breast carcinogenesis. Conditions that increase estrogen-to-androgen ratios, such as obesity, cirrhosis, testicular dysfunction, or estrogen therapy, promote ductal proliferation and malignant transformation [4,5]. Reduced androgen activity from orchitis, cryptorchidism, or orchiectomy may also contribute [5]. Lifestyle factors, including alcohol use, obesity, and physical inactivity, have been associated with increased risk, though evidence is variable [6].

Environmental exposures supplement this risk profile. Prior chest irradiation, including radiation historically used for gynecomastia, is associated with up to a seven-fold increase in MBC risk [5]. Occupational exposure in steel/rolling mills or to organic solvents (e.g., trichloroethylene) has also been implicated [5]. Despite extensive investigation, many associations remain inconclusive due to the rarity of MBC [4,6]. Overall, while MBC shares several risk factors with female breast cancer, particularly hormonal and hereditary influences, it displays a distinct biological profile, with higher rates of hormone-receptor positivity and lower HER2 expression [6]. These features have implications for both prognosis and therapeutic decision-making, underscoring the importance of genetic counseling and testing in all male patients [7].

Differential diagnosis

The differential diagnosis of EPC with a mixed invasive component in a male patient includes benign, atypical, malignant, and metastatic lesions. Accurate diagnosis requires careful correlation of clinical, radiologic, histologic, and immunophenotypic findings.

Gynecomastia, the most common male breast lesion, presents with subareolar thickening and shows florid ductal hyperplasia with stromal edema progressing to fibrosis [2,8]. While epithelial proliferation may be seen, gynecomastia lacks the complex, branching fibrovascular cores and cytologic atypia characteristic of EPC [2,8]. Atypical papillary hyperplasia may develop, particularly in men receiving estrogen therapy, but these lesions maintain an intact myoepithelial layer, unlike EPC, where myoepithelium is absent [2,8].

Benign papillary lesions, such as intraductal papilloma, exhibit fibrovascular cores lined by dual epithelial and myoepithelial layers [1,2]. Myoepithelium, which can be highlighted with p63, calponin, or smooth muscle myosin heavy chain, is the key feature distinguishing papilloma from EPC [1,2].

Malignant papillary lesions include solid papillary carcinoma (SPC) and IPC. SPCs form solid nests, may display neuroendocrine differentiation, and lack EPC’s encapsulated cystic architecture [1,2]. IPC, by definition, demonstrates overt stromal invasion and lacks a capsule, often eliciting stromal desmoplasia [1,2]. In EPC with invasion, each invasive component (e.g., NST, papillary carcinoma) must be separately characterized.

Metastatic tumors are a critical consideration in men. Metastatic prostate adenocarcinoma may mimic primary breast carcinoma and can express ER and AR [2,8]. Prostate-specific antigen (PSA) or prostate-specific acid phosphatase (PSAP) positivity supports prostatic origin, although PSA expression can occasionally be seen in breast tumors-usually after prolonged hormonal therapy [2,8]. The presence of DCIS or characteristic invasive patterns strongly supports a primary breast origin [2,8].

Metastatic thyroid carcinoma (papillary, follicular, or medullary) can resemble papillary or apocrine breast lesions; however, TTF-1 (SPT24 clone) and PAX8 positivity support thyroid origin, while breast carcinomas are typically negative [1,2].

Metastatic gastrointestinal adenocarcinoma, especially from the colon or rectum, may present as a mucinous breast mass. CDX2 and CK20 positivity favor gastrointestinal origin, whereas breast primaries are usually CDX2-negative [1,2].

Table 1 summarizes the key distinguishing features among these entities. 

**Table 1:** Differential diagnosis of EPC with an invasive component in the male breast. ER, estrogen receptor; PR, progesterone receptor; HER2, human epidermal growth factor receptor 2; ME, myoepithelial; PSA, prostate-specific antigen; PSAP, prostate-specific acid phosphatase; AR, androgen receptor; TTF-1, thyroid transcription factor-1; PAX8, paired box gene 8; CDX2, caudal-type homeobox 2; CK20, cytokeratin 20; EPC, encapsulated papillary carcinoma

Entity	Histopathologic Features	Myoepithelium	Immunohistochemistry
Gynecomastia	Florid ductal hyperplasia, stromal edema → fibrosis	Present	Not required
Intraductal papilloma	Fibrovascular cores with dual epithelium	Present	p63+, calponin+
Atypical papillary lesions	Mild atypia, limited papillary proliferation	Present (patchy)	Myoepithelial markers positive
Encapsulated papillary carcinoma	Papillary carcinoma within fibrous capsule, possible invasion	Absent	ER/PR+, HER2-low, ME markers negative
Solid papillary carcinoma	Solid nests with fibrovascular cores ± neuroendocrine features	Often absent	Synaptophysin+, chromogranin+
Invasive papillary carcinoma	Infiltrative papillary/cribriform growth	Absent	ER/PR+, Myoepithelial markers negative
Metastatic prostate cancer	A variable may mimic breast carcinoma	Absent	PSA+, PSAP+, AR+
Metastatic thyroid carcinoma	Tall-cell, follicular, or neuroendocrine morphology	Absent	TTF-1+, PAX8+
Metastatic gastrointestinal adenocarcinoma	Mucinous or gland-forming carcinoma	Absent	CDX2+, CK20+

Treatment considerations

Treatment of male breast cancer generally parallels recommendations for female breast cancer, as male-specific clinical trials remain limited [9,10]. In practice, mastectomy with axillary staging is the most common approach, followed by adjuvant therapy tailored to tumor biology and stage [9,10].

Surgery remains the primary treatment modality. Modified radical mastectomy is most frequently performed, although breast-conserving surgery may be appropriate in selected cases [9,10].

Endocrine therapy is central to the management of hormone-receptor-positive MBC [9,10]. Tamoxifen is the standard first-line agent, as aromatase inhibitors are less effective in men unless combined with a gonadotropin-releasing hormone (GnRH) analog to suppress testicular estrogen production [9,10].

Radiotherapy is recommended for post-mastectomy patients with high-risk features such as nodal involvement, large tumor size, or close margins. Retrospective studies suggest a survival benefit in men receiving adjuvant radiotherapy [9,10].

For metastatic MBC, systemic therapy aligns with female breast cancer guidelines: endocrine therapy for hormone-receptor-positive disease, HER2-targeted therapy when indicated, and chemotherapy or immunotherapy based on tumor characteristics and disease burden [9,10].

Because of the higher prevalence of BRCA2 and other pathogenic germline variants, routine referral for genetic counseling and testing (including BRCA1, BRCA2, PALB2, and others as appropriate) is strongly recommended for all men diagnosed with breast cancer [9,10].

## Conclusions

EPC of the male breast is an uncommon entity, and its occurrence with an associated mixed invasive component is exceptionally rare. This case highlights the importance of maintaining high suspicion when evaluating male breast masses, as clinical and radiologic features may overlap with benign lesions or metastatic disease. Accurate diagnosis requires comprehensive histopathologic assessment supplemented by immunohistochemistry to distinguish EPC from other papillary and invasive breast carcinomas, as well as from metastases to the breast.

Given the predominance of hormone-receptor-positive tumors in men, endocrine therapy remains central to management, while surgery and radiotherapy are tailored according to tumor stage and risk factors. The considerable contribution of hereditary cancer syndromes underscores the need for routine genetic counseling and germline testing in male patients.

This case reinforces the value of multidisciplinary collaboration among radiologists, pathologists, surgeons, medical oncologists, and genetic specialists to ensure precise diagnosis and individualized treatment planning. Continued reporting of rare male breast carcinoma subtypes such as EPC with mixed invasive components is essential to advance understanding and guide future clinical management.
